# Harnessing p53 for Proximity Killing

**DOI:** 10.3390/ijms27135725

**Published:** 2026-06-25

**Authors:** Joanna E. Zawacka

**Affiliations:** Cancer Center Karolinska, Theme Cancer, Karolinska University Hospital, 171 64 Stockholm, Sweden; info@joannazawackalab.se

**Keywords:** p53, MDM2, MDMX, mutant p53, proximity, PROTAC, TAPTAC, RIPTAC

## Abstract

p53 tumor suppressor evolved as a critical player in navigating the response to environmental stresses such as DNA or oxidative damage and drives cell fate by governing life and death decisions. The p53 protein is encoded by the most commonly mutated gene in human cancers. *TP53* gene mutations are associated with worse prognosis and refractory and relapsed disease. The most prevalent mutations are of the missense type and often result in disruption of the DNA-binding capacity and transcription activity. In healthy cells, p53 protein is tightly regulated by its E3 ubiquitin ligase, MDM2 (HDM2), its own transcription target. Mutant p53, therefore, escapes the regulation by the negative feedback loop and is often found upregulated in cancer cells. The efforts to exploit wild-type and mutant p53 for precision oncology have been ongoing in the last two decades yet have not been successful. A recently reported strategy to target *TP53*-mutant cancers leverages induced proximity, utilizing the high cellular abundance of mutant p53 as a scaffold to concentrate a small-molecule inhibitor against an essential survival protein. This strategy relies on the Regulated Induced Proximity TArgeting Chimera (RIPTAC). Given the recent FDA approval of the first chimeric drug, vepdegestrant, killing by proximity might turn out to be a promising medical advancement for precision oncology.

## 1. Introduction

p53 tumor suppressor, an intrinsically disordered protein of unresolved tertiary structure, and p53 functionality is ablated in all human cancer cases. The lack of a crystal structure of the full-length protein significantly hinders structure-based drug design and development. Current drug discovery approaches have fallen short in identifying p53-targeting molecules which are both specific and selective and display low-to-moderate side effects in clinical trials [1,2]. Missense mutations in *TP53* are the most prevalent aberrations affecting protein functionality. They disrupt p53 DNA-binding activity, are considered to drive the gain of new oncogenic functions to the altered protein and underlie poor prognosis. To date, no specific p53-targeting molecule has been approved by the Food and Drug Administration due to the late-phase failures [2,3].

In healthy cells, p53 activity and stability are maintained by post-translational modifications. Protein abundance is tightly controlled by protein turnover, largely mediated by MDM2-dependent ubiquitination and proteasomal degradation. Because *MDM2* is also a p53 target gene, the two proteins form a tightly regulated negative feedback loop. On average, the half-life of wild-type p53 under physiological conditions is reported to be 2–12 min, but can range up to 20 min depending on the tissue [4,5,6]. Contrary to the wild-type protein, mutant p53 proteins, defective in transcriptional activity, largely escape regulatory control by MDM2. This evasion is further reinforced by binding to chaperones like HSP70, enabling massive accumulation of mutant p53 proteins in cancer cells [7].

In human cancers, p53 is inactivated either by events leading to rapid protein turnover, like the amplification of the *MDM2* oncogene or by mutations in the *TP53* gene, ablating its ability to bind the canonical DNA sequence reviewed in [8]. Given that the restoration of p53 regresses established tumors in vivo [9], strategies aiming at reestablishment of the p53 pathway, either through targeting p53/MDM2/MDMX interactions or conformational recovery of the misfolded, mutated p53 protein, have been extensively studied for over two decades.

Compounds resembling the p53 amino acid triad of Phe^19^ (F19), Trp^23^ (W23), and Leu^26^ (L26), responsible for the p53/MDM2 interactions, belong to the group of nutlins. They are cis-imidazoline derivatives that occupy the pocket on MDM2 in place of p53’s Phe19, Trp23, and Leu26 residues. Thus, nutlins and their analogs efficiently prevent the protein–protein interactions. Several nutlin derivatives have been or are currently studied in clinical trials in cancer patients; however, the safety and efficacy remain to be elucidated due to the failures observed in Phase III studies.

The most advanced approaches aiming at harnessing mutant p53 for precision oncology are exemplified by APR-246/eprenetapopt and arsenic trioxide/Trisenox. Both drugs target cysteines located in the DNA-binding domain of p53, yet have distinct target cysteine residues [10,11]. MQ (methylene quinuclidinone), an active form of APR-246, is a thiol-reactive alkylating agent, refolding mutant p53 and covalently targets surface-exposed, or close to surface cysteines 124, 182, 229, 275 and 277 as shown in example for the DNA-unbound R273H core domain [12]. On the other hand, ATO, in its highly thiophilic form (As(OH)_3_), binds to the cysteine triad at positions 124, 135 and 141 and induces an allosteric shift resulting in the change of the conformation [11]. Due to the high affinity of both MQ and As(OH)_3_ for thiols (sulfhydryl groups, -SH), the specificity of these drugs may dependent on the cellular concentration of mutant p53 in cancer cells. Thus, a new strategy has recently been established and brought to the clinical trial setting, which converges on targeting a novel pocket in Y220C mutant p53 with a small molecule, as described in detail below. The novel chimeric drug exploits the PMV6 molecule, a highly specific Y220C mutant p53 binder. PMV6 is currently undergoing Phase II clinical trials and serves as a first-in-class therapy designed to exhibit high specificity towards a defined p53 missense mutation.

## 2. Targeting Cancer Vulnerabilities by Rationally Designed Proximity

One of the newest promising approaches in inactivating MDM2 oncogene is through employing proximity-induced degradation using rationally designed proteolysis-targeting chimeras (PROTACs), as recently reported [13]. In that study, Adams et al. described the design and synthesis of the MDM2-PROTAC, YX-02-030, by altering the clinically available MDM2 inhibitor, an analog of nutlin, RG7112. The rational design led to the replacement of the 3-(methylsulfonyl)-propyl tail on the piperazine motif of RG7112 with an acetic acid. This resulted in the generation of the RG7112D molecule, which was next coupled to VHL-amine, of the VHL E3 ligase recruiting ligand. The MDM2-PROTAC binds with a high affinity to MDM2 and VHL, forming a ternary complex to induce selective degradation of MDM2.

**Figure 1:**
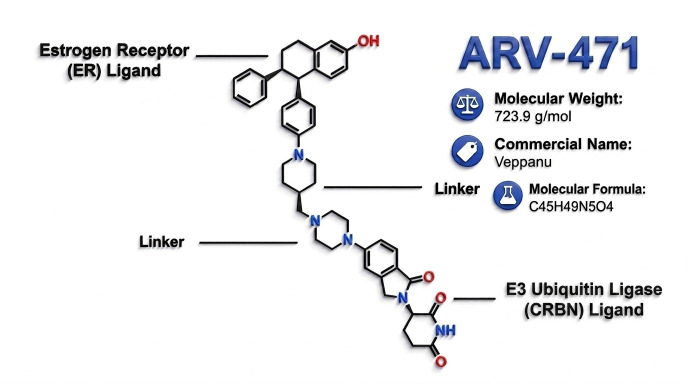
Chemical structure of the FDA-approved PROTAC, vepdegestrant/Veppanu. Prepared with FigureLabs.ai.

On average, PROTACs have molecular sizes ranging from 700 to 1200 Da and are composed of a targeting warhead (POI Ligand) of about 300–400 Da, an E3 ligase recruiter of 250–450 Da and a chemical linker of 100–250 Da, making them quite large when compared with small-molecule kinase inhibitors; see Figure 1 (reviewed in [14]). As of 1 May 2026, the first-in-class, oral PROTAC targeting the ER oncogene for the treatment of adult patients with ER-positive (+), HER2-negative (−), and solely ESR1-mutated advanced or metastatic breast cancer has been approved by the Food and Drug Administration. The molecule was designed to induce the targeted degradation of the estrogen receptor (ER). VEPPANU™ (vepdegestrant/ARV-471) is a single small molecule composed of an estrogen receptor ligand-binding domain that binds specifically to ERα and the E3 ligase-binding moiety that binds specifically to the intracellular E3 ubiquitin ligase enzyme Cereblon (CRBN) and is 724 Da in size (Figure 1). Vepdegestrant displays a critical milestone as the first FDA-approved heterobifunctional protein degrader. By rationally designed enforcement of the proximity between the estrogen receptor and the cereblon E3 ligase, the drug induces robust proteasomal degradation, more than doubling progression-free survival compared to standard-of-care fulvestrant in the mut*ESR1* patients [15].

This landmark FDA approval establishes a critical regulatory precedent, paving the way for the broader translation of heterobifunctional chimeras into precision oncology. Consequently, PROTAC pipelines continue to expand, targeting a diverse range of historically challenging oncogenic drivers, including KRAS, EGFR, ALK, BRAF, and c-MYC. Relevantly, on 9 June 2026, the Food and Drug Administration has cleared AMX-883, a potent, orally bioavailable DCAF16-dependent degrader of BRD9, for acute myeloid leukemia (AML). It will be tested in a phase 1 monotherapy dose-escalation and optimization trial and will enroll patients with relapsed or refractory AML and high-risk myelodysplastic syndrome [16].

Unlike PROTACs, which are designed to recruit E3 ubiquitin ligases to actively eradicate targeted oncogenes via the proteasome, RIPTACs do not deplete or degrade their targets. Instead, this novel strategy functions through mechanism-based target accumulation, physically tethering an overexpressed tumor protein to an essential intracellular survival protein (such as RNA Polymerase II) [17]. This cooperative binding tethers both partners within a stable, non-functional ternary complex that sterically hinders the survival protein, effectively neutralizing its vital cellular function to force selective cancer cell death. By relying on the utter abundance of a tumor antigen rather than its downstream signaling or active enzymatic degradation machinery, RIPTACs are intrinsically less susceptible to the traditional mutational resistance mechanisms that frequently compromise standard targeted therapies [17].

## 3. Harnessing p53 for Proximity Killing

**Figure 2:**
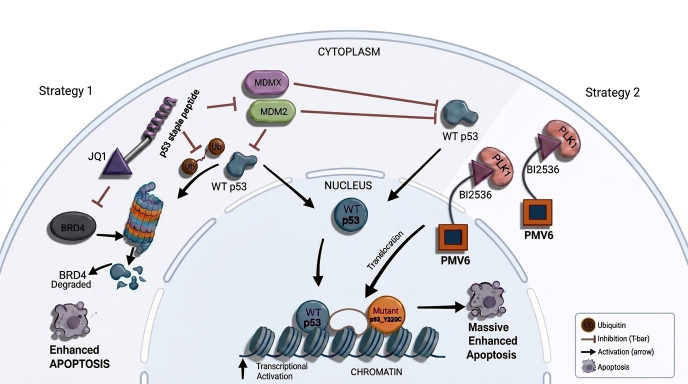
Harnessing p53 for proximity killing. Strategy 1: A strategy using tri-functional PROTAC-derived chimera TAPTAC, which inhibits MDM2 and MDM4 by a hyper-stable, cell-permeable p53-derived staple peptide and uses the JQ1 molecule, a potent inhibitor of oncogenic BRD4, for induction of massive apoptosis. Strategy 2: p53-01, a novel RIPTAC which exploits highly abundant mutant p53^Y220C^ and has a PMV6 binder to drive the accumulation of the essential gene PLK1 in the nucleus by attaching it to BI2536, which inhibits proliferation and induces cell death. Prepared with FigureLabs.ai.

A novel tri-functional PROTAC has been developed to induce apoptosis in wild-type p53 cancer cells. A contemporary study by Bird et al. established a triple-action proteolysis targeting chimera (TAPTAC) utilizing stapled peptide technology that efficiently reactivates the biologically active alpha-helical structure of the investigated peptide while enabling proteolytic resistance [18]. The authors employed a cell-permeable p53 stapled peptide, which binds both the MDM2 and MDM4(X) oncogenes, making it particularly efficient in restoring p53 activity in cancer cells (Figure 2). Molecules found to have a dual action, thus inhibiting p53/MDM2/MDM4 interactions, were recently reported [19] and some are in clinical trial studies. These include sulanemadlin (ALRN-6924), a first-in-class, cell-permeable stapled peptide [20]. The second component of the TAPTAC molecule includes JQ1, a BET inhibitor which interacts with the BD1 domain of BRD4 (BRD4BD1) and induces its degradation. The resulting 3-in-1 approach delivers a highly precise killing degrader which simultaneously restores wild-type p53 and blocks the critical regulator of oncogenic pathways (Figure 2).

RIPTACs are another example of molecules that allow for harnessing proximity for therapeutic purposes. They are designed so that the heterobifunctional compound facilitates a stable ternary complex between a target which is selectively expressed in tumor cells and a ubiquitously expressed protein essential for cell survival. The rationale behind RIPTACs has been recently applied by Sadagopan et al. [21] to target mutant p53-loaded cancer cells.

p53 mutants are highly abundant in mut*TP53* mono- and biallelic cancer cells as they escape the p53-MDM2 negative regulatory feedback loop. Of late, Sadagopan et al. showed in a genome-wide analysis of CRISPR dependencies that this abundance of mutp53s appears to be the only genetic or proteomic distinction between *TP53*-mutant and *TP53*-wild-type cancer cells and thus provides a unique therapeutic opportunity. To take advantage of the mutp53 abundance, the team first generated HEK293-derived and Calu-1-derived cell lines overexpressing Halo-tag mutant p53 (Halo–p53-R273H (FL)–mCherry) or wild-type p53 (Halo–p53 WT (FL)–mCherry–2A–mTagBFP2-V5). Next, the team treated the cells with a novel, bifunctional molecule liganding with the tagged mutp53s and containing PLK1 inhibitor, Halo-PEG2-BI2536. PLK1 was identified as a highly essential yet low-abundance protein by comparing functional genomics data from the Dependency Map (DepMap) with absolute protein quantification from OpenCell. It is recognized as an oncogene, critical for mitotic progression, and the BI2536 inhibitor, used in the study, was shown to induce G2/M arrest. Bifunctional molecules containing mutp53-tag and PLK1 inhibitor selectively inhibited the proliferation of *TP53*^R273H^ and *TP53*^Y220C^-mutant cells with a good therapeutic window, which was more prominent in Calu-1 cells.

The Y220C mutation accounts for around 1.8% of all somatic mutations, and p53^Y220C^ is exploited for improved cancer therapy. Thus, next, the authors tested the bifunctional binder containing the PMV6 molecule, rezatapopt, owned by PMV Pharma, which induces thermostabilisation of p53^Y220C^ core domain by 8 °C and binds to the unique pocket in p53 created by the Y220C substitution. The activity of the bifunctional molecule containing PMV6 and BI2536 (annotated as p53-01) was assessed to be at EC_50_ = 1.4 μM, as reflected by the induction of mitotic arrest and apoptosis. Upon p53-01 treatment, PLK1 colocalized with p53^Y220C^ on chromatin (Figure 2). Authors speculated that such mislocalization could significantly contribute to the disruption of the function of PLK1 as a mitotic kinase and thus beyond simple steric blockade of its active site. The response in cells was mutp53^Y220C^-dependent, yet without reactivation of mutant p53 protein to wild-type protein and induction of p53 response program. Finally, the team, through linker optimization, generated the PMV6-C3-BI2536 molecule, which was more potent than p53-01 by >1.5 orders of magnitude as assessed in the competition assay (PMV6-C3-BI2536 EC_50_: 25 nM, p53-01 EC_50_: 850 nM). Thus, the bifunctionals targeting mutant p53 abundance and essential genes with the previously identified toxins might open up new avenues for the development of more selective and specific treatments for mut*TP53* cancers.

## 4. Conclusions

In summary, induced proximity killing has emerged as a promising frontier in precision oncology. While already well-established bifunctional molecules primarily serve as molecular glues to degrade key oncogenic drivers, next-generation chimeric modalities introduce fundamentally novel therapeutic paradigms. For instance, TAPTACs (triple-action proteolysis targeting chimeras) simultaneously target MDM2/MDMX and BRD4 via induced proximity, thereby synergistically reconstituting wild-type p53 signaling while degrading oncoproteins. Conversely, mutant p53-targeting RIPTAC harnesses the high intracellular abundance of mutant p53 protein, employing it as a structural scaffold to translocate and inhibit the essential survival gene product, PLK1. By systematically exploiting dual cancer vulnerabilities or turning cancer-specific mutant protein abundance into a lethal molecular trap, the recently developed proximity-based strategies might, in the future, offer advanced therapeutic options for precision oncology that may ultimately circumvent traditional resistance mechanisms.

## Data Availability

No new data were created or analyzed in this study. Data sharing is not applicable to this article.
